# Association between Area-Level Socioeconomic Deprivation and Prehospital Delay in Acute Ischemic Stroke Patients: An Ecological Study

**DOI:** 10.3390/ijerph17207392

**Published:** 2020-10-11

**Authors:** Hang A Park, Hye Ah Lee, Ju Ok Park

**Affiliations:** 1 Department of Emergency Medicine, Hallym University College of Medicine, Dongtan Sacred Heart Hospital, Chuncheon 18450, Korea; hangapark@hallym.or.kr; 2 Department of Epidemiology, School of Public Health, Seoul National University, Seoul 08826, Korea; 3 Clinical Trial Center, Mokdong Hospital, Ewha Womans University, Seoul 07985, Korea; khyeah@ewha.ac.kr

**Keywords:** stroke, socioeconomic status, prehospital delay

## Abstract

We analyzed the associations between area-level socioeconomic status (SES) and prehospital delay in acute ischemic stroke (AIS) patients by degree of urbanization with the use of an ecological framework. The participants were 13,637 patients over 18 years of age who experienced AIS from 2007 to 2012 and were admitted to any of the 29 hospitals in South Korea. Area-level SES was determined using 11 variables from the 2010 Korean census. The primary outcome was a prehospital delay (more than three hours from AIS onset time). Multilevel logistic regression was conducted to define the associations of individual- and area-level SES with prehospital delay after adjusting for confounders, which includes the use of emergency medical services (EMS) and individual SES. After adjusting for covariates, it was found that the area-level SES and urbanization were not associated with prehospital delay and EMS use was beneficial in both urban and rural areas. However, after stratification by urbanization, low area-level SES was significantly associated with a prehospital delay in urban areas (adjusted odds ratio (aOR) 1.24, 95% confidence interval (CI) 1.04–1.47) but not in rural areas (aOR 1.04, 95% CI 0.78–1.38). Therefore, we posit that area-level SES in urban areas might be a significant barrier to improving prehospital delay in AIS patients.

## 1. Introduction

Stroke remains one of the most devastating of all neurological conditions. Globally, it accounts for approximately 5.5 million deaths annually, with 44 million disability-adjusted life years lost. As a disease of aging, the prevalence of stroke is expected to increase significantly around the world [1]. The past decades have shown an increase in the use of thrombolysis, which has shown significant results in the breakdown of blood clots formed in blood vessels [2,3,4]. A main limitation for thrombolytic treatment is the narrow time window from symptom onset to needle. Even with the recent extension of the time window up to 4.5 h and the increasing use of the intraarterial approach, less than 5% of patients with acute stroke presently receive thrombolysis [5,6]. To improve this, it is necessary to minimize the time spent in the prehospital stage, that is, the prehospital delay. Research has shown that various patient-related factors such as diabetes, illiteracy, an unfavored social class, and living alone can cause prehospital delays [7]. When acute ischemic stroke (AIS) occurs, the patients could be unaware of the symptoms and may consider it unserious, and this may affect the required prompt treatment. In this case, public education is key to improving and sustaining the community’s knowledge of the early signs of stroke, especially for groups at the highest risk of stroke.

Among the many risk factors of prehospital delay, some researchers believe that low socioeconomic status is one of the causes [8,9]. Moreover, some studies have found a significant relationship between prehospital delay and individual socioeconomic status (SES) status [7,10,11]. Other studies have explored the relationship between neighborhood/community SES and prehospital delay in AIS patients [12,13] with varying results. A possible reason for the inconsistent results is the extent of urbanization. Urbanization implies a change in the economic, social, and cultural aspects of the society. It refers to the process of becoming urban; the movement of people or processes to urban areas; and an increase of urban areas, population, or process [14]. As the distribution of area-level SES differs between urban and rural districts, a stratified analysis by urbanization status is needed.

Thus, we used an ecological framework to compare the associations between area-level SES and prehospital delay among AIS patients based on the degree of urbanization. Data from different regions and institutions should be sufficiently collected to achieve urbanization stratification. However, such data is challenging to collect and manage. Moreover, indicators for measuring the level of deprivation in each area should be applied to reveal the effect of SES. In this study, the authors attempted stratification by using multicenter data, targeting the whole country. By using a valid deprivation index, we could measure the effect of SES reasonably. Moreover, as far as we know, this is the first study to apply a multilevel approach to access prehospital delay.

## 2. Materials and Methods

### 2.1. Study Setting

There are 17 administrative divisions in South Korea: nine provinces (including Jeju Special Self-Governing Province), six metropolises, one “special city,” and one “special self-governing city.” These divisions are subdivided into several smaller entities, including cities (si), counties (gun), and districts (gu). Provinces, the highest administrative category in Korea, are divided into counties. In the case of an urbanized area with a population of over 50,000 among counties, it can be independent as a city. Additionally, among them, cities with a population of over 500,000 are divided into districts. Therefore, in this study, districts of large cities, undivided cities, and other counties were applied at the same level of regional units. The determinant for urban area, based on the 2010 population census, is districts within a city having a population greater than 500,000. Finally, the country was divided into 248 regional units, of which 107 units belong to the urban area category and 141 units belong to the rural area category. For each unit, the area-level SES index, which will be described later in this section (2.3.1. Area-Level Socioeconomic Index), was measured.

South Korean emergency medical services (EMS) are provided by the National Fire Agency through a Basic Life Support (BLS) single-tier system, a system that dispatches the same level of EMS regardless of the patient’s condition. Thus, the administration of intravenous thrombolysis by an EMS provider before hospital arrival is illegal [15].

### 2.2. Study Participants

The Cardiovascular Disease Surveillance (CAVAS) project is a nationwide prospective stroke registry sponsored by the Korean Centers for Disease Control [15]. Patients were accepted in the study if they were diagnosed with acute stroke after a neurological examination, brain computed tomography, or magnetic resonance imaging on visiting the emergency room with symptoms of acute stroke such as motor weakness, sensory changes, or consciousness changes.

**Figure 1:**
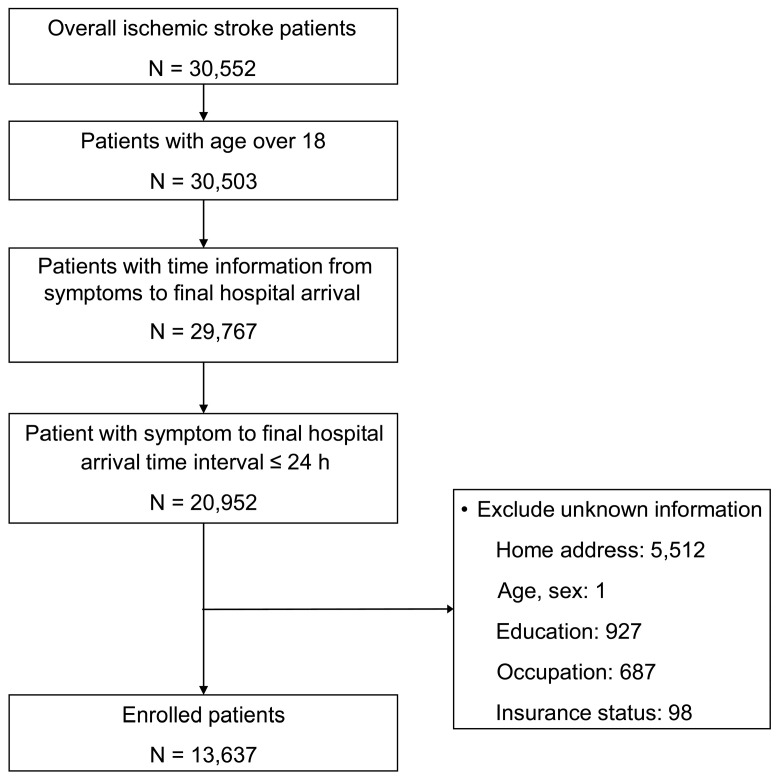
Flow chart showing patient recruitment according to the inclusion and exclusion criteria.

The study included all adult patients (aged 18 years and above) to whom the International Classification of Disease-10th Revision code I63 or I64 was assigned on emergency department discharge between 1 November 2007 and 31 December 2012. Patients whose stroke onset or hospital arrival time data were missing were excluded, as were those who arrived 24 h after the onset of symptoms. Those without data on EMS use, address, age, sex, or individual-level SES were excluded, as well (Figure 1).

### 2.3. Data Collection

#### 2.3.1. Area-Level Socioeconomic Index

Area-level SES was based on a deprivation index created by sampling 10% of the data from the 2010 Korean population census, recorded at the district level by the Korean National Statistics Office [16]. Most deprivation variables were very similar to those used previously [17] (Supplementary Table S1). Each variable was Z-score standardized, and the values were then combined to calculate deprivation indices at the district level [18]. The median district deprivation index nationwide was −1.78 (range: −16.67 (least deprived) to 18.03 (most deprived); the standard deviation (SD) was 7.96). The entire area was divided by thirds in the order of deprivation index. The regions with the highest deprivation index were sequentially divided into deprived, middle, and affluent groups.

All the regions were stratified into urban and rural at the provincial administrative level. If the province where the patient belonged had more than 500,000 people according to the 2010 census population, it became an urban area. In the urban areas, there were 47 (36.2%) districts belonging to the affluent category, 39 (36.4%) middle, and 21 (19.6%) deprived. In the rural areas, there were 20, 22, and 99 (14.2%, 15.6%, and 70.2%) districts, respectively. The patients’ characteristics related with urbanization are shown in Supplementary Table S2.

#### 2.3.2. Individual-Level Socioeconomic Variables

The CAVAS-derived education level and occupation status data were used to denote individual SES. The education level was divided into high school graduates and non-high school graduates. Occupations were then classified using the 10 major types of the Korean Standard Classification of Occupations based on the International Standard Classification of Occupations [19]. We divided the 10 occupation types into four classes: manual workers (sales staff; skilled, craft, trade, and assembly workers; equipment and machine operators), non-manual workers (managers, professionals, clerks, and service workers), other workers (armed forces personnel, students, and homemakers), and economically inactive (unemployed and retired). As a measure of current economic status, unemployed persons and retirees were classified together as economically inactive. This is because it was previously reported that these two groups tend to have similar health statuses [20]. Insurance status was classified as coverage by the National Health Insurance and Medicaid.

#### 2.3.3. Patient Characteristics

We retrieved age, sex, and clinical information, times of AIS onset and hospital arrival, EMS use, and interhospital transfer status from the CAVAS registry. We then divided the patients into three age groups (19–40, 40–65, and >65 years). The onset time was divided into day (6 a.m. to 6 p.m.) and night (6 p.m. to 6 a.m.) (Table 1).

#### 2.3.4. Outcome Variables

The primary outcome was a prehospital delay (more than three hours from AIS onset time). The AIS onset time corresponds to the time at which the patient or caregiver first noticed symptoms or to the last known normal time if symptoms were not witnessed. According to the treatment guidelines published in 2018 [21], IV alteplase (thrombolytics) treatment is recommended for selected patients who can be treated within 4.5 h of ischemic symptom onset or when the patient was last seen well. In addition, it is recommended that a primary goal of achieving door-to-needle times of within 60 min in ≥50% of AIS patients treated with IV alteplase should be established in hospital care. Considering the reasonable time required for the initial evaluation (such as physical exam, laboratory, and radiology tests), thrombolytic treatment can be performed following the above guidelines only if patients arrive within 3 h from the onset of symptoms. Therefore, in this study, the outcome was set based on 3 h.

### 2.4. Statistical Analysis

Summary statistics were presented as median with interquartile range for non-normal distributed variables and as frequency with proportion for categorical variables. Differences in prehospital time intervals by urbanization status or area-level SES were assessed using the Wilcoxon rank-sum test and Kruskal–Wallis test. We used the chi-squared test to compare the groups in terms of area-level SES. Subsequently, we evaluated hierarchically to determine the effect of individual SES and area-level SES on prehospital delay using multilevel multivariable logistic regression. A random intercept-only model (i.e., basic model) was used to analyze the area-level variance in SES, and intraclass correlations were calculated. The associations of individual SES parameters with prehospital delay were assessed and specified as Model 1. Model 2 was evaluated by adding area-level SES variables to Model 1. Furthermore, the characteristics of patients significantly related to prehospital delay in univariate analysis were included in Model 3. Model 3 was further evaluated by stratification according to urbanization. Stratification by urbanization was carried out for two reasons: the interaction terms of area-level SES and urbanization were significant when the fitness was tested (*p* = 0.0014), and there were differences in the variables of various prehospital stages depending on urbanization. Statistical significance was decided with 95% confidence intervals (CIs). The multilevel logistic regression models were generated using the GLIMMIX procedure of SAS ver. 9.4 (SAS Institute, Cary, NC, USA).

### 2.5. Ethical Approval

The present study was reviewed and approved by the Institutional Review Board of Seoul National University Hospital with a waiver of consent in emergency medicine research (approval no. 1012-134-346).

## 3. Results

### 3.1. Participants’ Baseline Characteristics

Out of 30,552 adult AIS patients, 13,637 were qualified to be included in this study (Figure 1). Of this sample, 5174 lived in rural areas, while 8463 lived in urban areas. Table 1 lists patient characteristics by area-level SES group. About 66% of the patients were over 65 years of age, and 57.4% were female. A total of 4405 patients (32.3%) used EMS; its use was highest in the middle group and lowest in the deprived group (36.3% and 27.6%, respectively).

### 3.2. Correlation between Area-Level and Individual-Level SES

Of all participants, 60.3% had not completed high school, 55.6% were unemployed, and 26.8% were manual workers. The affluent group included more high school graduates and non-manual workers. The proportion of Medicaid patients was highest in the deprived group (Table 1).

### 3.3. Associations between Area-Level SES and Prehospital Delay

Among all the patients, 59.4% experienced a prehospital delay. The median time from AIS onset to hospital arrival was 4.3 h (interquartile range (IQR) 1.7–10.4), 4.0 h (IQR 1.4–10.0), and 4.6 h (IQR 2.0–10.8) for the affluent, middle, and deprived groups, respectively (*p* < 0.001) (Table 2). Of the patients who used EMS, the median time from symptom onset to when EMS was called was 52 min (IQR 10–261), 60 min (IQR 12–282), and 67 min (IQR 15–349) for the affluent, middle, and deprived groups, respectively (*p* = 0.04).

Table 3 shows that area-level SES was significant in the basic random effects model (*p* < 0.001). This means that area-level SES was significantly associated with prehospital delay. Considering the difference in the variance of random effects, individual-level SES explained 3.0% of the variance in prehospital delay (basic model and Model 1), and area-level SES accounted for an additional 6.8% of the variance in prehospital delay (Model 1 and Model 2).

The odds for a prehospital delay in the deprived group were 1.28 times higher than the odds in the affluent group in the unadjusted model (odds ratio (OR): 1.28; 95% CI: 1.10–1.48). However, the difference was not significant after adjusting for all covariates (adjusted OR (aOR): 1.03; 95% CI: 0.89–1.20). A higher education level predicted a low probability of prehospital delay in the unadjusted model (OR: 0.91; 95% CI: 0.85–0.98) but not in the final model (aOR: 0.93; 95% CI: 0.85–1.32) (Table 3).

### 3.4. Stratified Analysis by Urbanization Status

**Figure 2:**
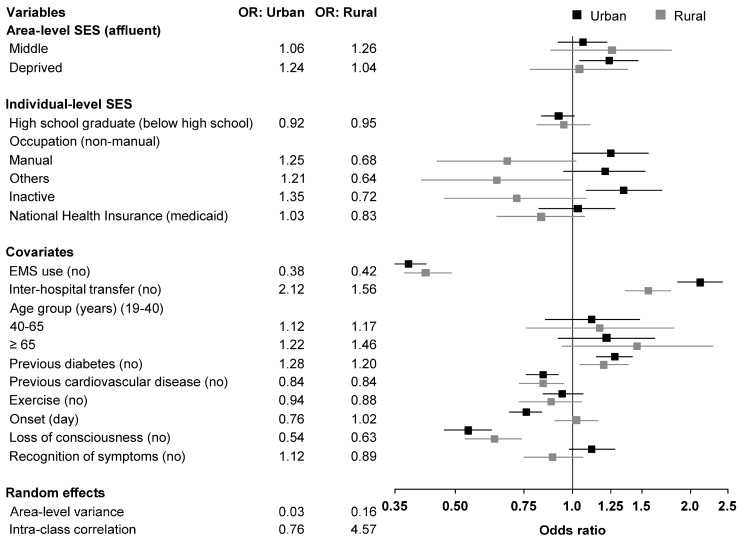
Odds ratio of prehospital delay stratified by urbanization status.

In urban areas, the odds for a prehospital delay were 1.24 times higher in the deprived group (aOR: 1.24; 95% CI: 1.04–1.47) than those in the affluent group, and the odds among economically inactive patients were 1.35 times higher than those among non-manual workers (aOR: 1.35; 95% CI: 1.08–1.69). In rural areas, we found no significant association between area-level SES and prehospital delay. The “other” occupational group was significantly more likely to experience prehospital delay compared to non-manual workers (aOR 0.64; 95% CI: 0.41–0.99) in rural areas (Table 4 and Figure 2). EMS use was an obvious advantage for AIS patients to arrive at the hospital in time in both urban (aOR 0.38; 95% CI: 0.35–0.42) and rural areas (aOR 0.42; 95% CI: 0.37–0.49). In other words, interhospital transfers resulted in prehospital delays in both areas.

## 4. Discussion

In this study, we examined whether area-level SES was associated with prehospital delay for AIS patients according to their degree of urbanization. Patients from deprived areas experienced approximately a 36 min longer delay compared to those from affluent areas. The association between area-level SES and prehospital delay differed according to the degree of urbanization; those classified as deprived in the urban areas faced a higher risk of delay than those in other area-level SES groups. However, there was no difference in the risk of prehospital delay between the deprived and the affluent in rural areas. A unique feature of this study is its use of a deprivation index that determines the SES of residential areas based on various factors. Given that SES is a complex metric that comprises multiple social factors, we tried to collect various types of information that reflect individual- and area-level SES to obtain an accurate representation of the SES level. Through the multilevel analysis, which included both the individual- and area-level SES, we aimed to determine the amount of variance in prehospital delay explained by both these types of SES.

Prehospital delay is one of the major concerns of AIS treatment, and studies of cardiovascular care have reported some inequality in service and treatment provision for those in low-SES groups [9]. In AIS, many studies have shown differences in community or individual SES for diagnostic tests such as computed tomography (CT) scan and magnetic resonance imaging (MRI), thrombolytic treatment, or rehabilitation [9,22], but surprisingly, few studies are available on the relationship between prehospital delay and SES. One study in the U.K. found that patients from the affluent group according to the Carstairs DepCat scores [23] reached hospitals sooner following a stroke [8], and these results are consistent with our findings. The previous study by Macleod et al. had a major limitation in terms of generalization because it focused only on patients who visited one hospital in Edinburgh. However, our study included various urbanization areas across the country, and the results after the stratification of urbanization will allow for generalizing these findings to other countries. The results of our study revealed that area-level SES-related disparities in prehospital delay were greater in urban areas than in rural areas, even after adjusting for individual SES, EMS use, and other factors such as diabetes, history of cardiovascular diseases, and onset time. This finding is in contrast with the Kleindorfer et al. study, which revealed that those living in relatively deprived areas did not appear to experience delayed access to acute stroke care to a clinically significant extent [13]. As shown in Table 4, the aOR of middle-SES and deprived-SES groups in rural areas was not statistically significant. In other words, the probability of prehospital delay in rural areas was not significantly different between different SES groups. However, in urban areas, it was statistically significant in that the deprived group had a 1.24 times higher probability of prehospital delay than the affluent group. Before stratification, the area-level SES appeared to be unrelated to prehospital delay, but after stratification, the area-level SES seemed to be related to the prehospital delay, and we assumed that this difference was due to the difference in the composition of area-level SES in rural and urban areas. When considering area-level SES, due to the gap in SES in urban areas, we expected that the imbalance for the deprived group would occur more in urban areas than in rural areas. The results of this study found that the risk of prehospital delay was not high at a specific level classified according to the criteria of area-level SES. Instead, it seemed that the greater the gap of SES with surrounding areas, the higher the risk of prehospital delay.

As shown in Supplementary Table S2, the characteristics of patients in urban and rural areas show significant differences. In rural areas, for example, the proportion of patients with low individual SES is significantly higher. In other words, low individual SES can increase the risk of stroke. Meanwhile, the rate of using EMS in rural areas is high, so it is expected that this would lead to a good prognosis.

Interestingly, among patients who used EMS, the time of the call from the onset of symptoms was significantly longer in the deprived group than in the affluent group (Table 2); however, it was found that the difference in the time of the call from the onset of symptoms between urban and rural areas was not significant (Supplementary Table S2). These findings suggest that the perception of symptoms of AIS differs by SES rather than by urbanization. Studies have shown that awareness of stroke is higher in the urban population than in the rural population, highlighting the need for stroke awareness campaigns in rural areas [24,25]. However, our study showed that strategies to increase stroke awareness should be based on SES rather than on urbanization.

For patients who were transferred from another hospital, even if the time from symptom onset to arrival at the first hospital was shorter for patients in rural areas, the time from symptom onset to arrival at the final hospital was significantly longer for patients in rural areas compared to that for those in urban areas (Supplementary Table S3). For patients who used EMS, while the time to call from symptom onset was similar for patients in both rural and urban areas, the time from the call to the hospital was significantly longer for patients in rural areas compared to that for those in urban areas (Supplementary Table S3). These results suggest that the distance to the hospital was related to prehospital delays in rural areas. Given that the transportation time to the comprehensive stroke unit takes longer in rural areas, EMS use and interhospital transfer might have a greater effect on prehospital delays for patients in rural areas. Meanwhile, urban areas have better access to final hospitals. Thus, it is likely that there is an association between area-level SES and prehospital delays.

EMS use and interhospital transfer are crucial for reducing prehospital delays [15,26,27,28]. In this study, EMS use significantly reduced prehospital delays, independent of the urbanization status and area-level SES. The rate of EMS use was lower in the deprived group than that in the affluent group. However, the time from EMS notification to EMS arrival appeared to be shorter in deprived areas (Table 2), indicating that EMS accessibility was not low in the deprived group in Korea.

## 5. Strength and Limitation of the Study

The strengths of this study were that it used a nationwide dataset, included patients regardless of EMS usage, was stratified by urbanization, and focused on the gap of SES as well the absolute level of SES.

Like most studies, this study has its limitations. First, we excluded 7823 patients because of missing data, which might have introduced sampling bias. However, an analysis of the home addresses of the 1802 patients whose addresses were recorded but whose other data were missing revealed similar proportions in different area-level SES groups (affluent group, 607 (33.7%); middle group, 606 (33.6%); deprived group, 589 (32.7%)). Therefore, the exclusion of these patients did not significantly affect the results of this study. Finally, given the possible differences in social structure, education, and health services, the results may differ in other countries. Thus, further research is required to confirm this.

## 6. Conclusions

Area-level SES is not associated with a prehospital delay in AIS patients after adjusting for covariates. However, residing in a deprived urban area may increase prehospital delay, although this does not appear to be the case in rural areas. Area-level SES in urban areas might be a significant barrier to reducing prehospital delay in AIS patients. Finally, EMS use plays a crucial role in reducing prehospital delay, independent of urbanization status and area-level SES. EMS personnel must, therefore, consider potential preconceived ideas or biases for low-SES patients.

## Figures and Tables

**Table 1:** Characteristics of patients by area-level socioeconomic status (SES).

Variable	Total		Affluent		Middle		Deprived		*p*
	*N*	%	*N*	%	*N*	%	*N*	%	
	13,637		4489		4615		4533		
**Sex** (female)	7822	57.4	2637	58.7	2665	57.7	2520	55.6	0.01
**Age group** (years)									<0.001
19–40	352	2.58	141	3.14	126	2.73	85	1.88	
40–65	4290	31.5	1614	36.0	1471	31.9	1205	26.6	
≥65	8995	66.0	2734	60.9	3018	65.4	3243	71.5	
**Education level**									<0.001
Below high school	8228	60.3	2282	50.8	2615	56.7	3331	73.5	
≥High school graduate	5409	39.7	2207	49.2	2000	43.3	1202	26.5	
**Occupation type**									<0.001
Non-manual	565	4.14	226	5.03	220	4.77	119	2.63	
Manual	3656	26.8	1138	25.4	1052	22.8	1466	32.3	
Other	1834	13.4	626	13.9	739	16.0	469	10.3	
Inactive	7582	55.6	2499	55.7	2604	56.4	2479	54.7	
**Insurance type**									<0.001
National Health Insurance	12,953	95.0	4322	96.3	4373	94.8	4258	93.9	
Medicaid	684	5.02	167	3.72	242	5.24	275	6.07	
**Past medical history**									
Diabetes	3521	25.8	1224	27.3	1245	27.0	1052	23.2	<0.001
Hypertension	7985	58.6	2694	60.0	2760	59.8	2531	55.8	<0.001
Cardiovascular disease	2689	19.7	879	19.6	978	21.2	832	18.4	<0.001
Exercise (yes)	2673	19.6	972	21.7	984	21.3	717	15.8	<0.001
**Smoker**									0.12
No	8020	58.8	2646	58.9	2652	57.5	2722	60.0	
Former	2248	16.5	732	16.3	803	17.4	713	15.7	
Current	3369	24.7	1111	24.7	1160	25.1	1098	24.2	
**Alcohol consumption**									0.02
Never	9320	68.3	2998	66.8	3200	69.3	3122	68.9	
Yes	4317	31.7	1491	33.2	1415	30.7	1411	31.1	
**Onset**									0.03
Day	9105	66.8	3063	68.2	3037	65.8	3005	66.3	
Night	4532	33.2	1426	31.8	1578	34.2	1528	33.7	
**Loss of consciousness**	2237	16.4	654	14.6	723	15.7	860	19.0	<0.001
**Recognition of symptoms**	11,319	83.0	3662	81.6	3767	81.6	3890	85.8	<0.001
**EMS use**	9232	67.7	3014	67.1	2938	63.7	3280	72.4	<0.001
**Interhospital transfer**	4052	29.7	1086	24.2	1032	22.4	1934	42.7	<0.001
**Prehospital delay**	8096	59.4	2557	57.0	2699	58.5	2840	62.7	<0.001
**ED outcome**									<0.001
Discharge	264	1.94	78	1.74	112	2.43	74	1.63	
Transfer	417	3.06	85	1.89	109	2.36	223	4.92	
Admission	12,916	94.7	4314	96.1	4383	95.0	4219	93.1	
Death	27	0.20	7	0.16	6	0.13	14	0.31	
Unknown	13	0.10	5	0.11	5	0.11	3	0.07	
**Residence**									<0.001
Urban	8463	62.1	3637	81.0	3519	76.3	1307	28.8	
Rural	5174	37.9	852	19.0	1096	23.7	3226	71.2	

Abbreviations: ED—emergency department; EMS—emergency medical service. The *p*-values were calculated using the chi-squared test.

**Table 2:** Prehospital time intervals by area-level SES.

Variable	Total	Affluent	Middle	Deprived	*p*
**Total patients**					
Symptom onset to final hospital (hours)	4.3 (1.7–10.4)	4.0 (1.4–10.0)	4.2 (1.4–10.6)	4.6 (2.0–10.8)	<0.001
**Patients who were transferred**					
Symptom onset to first hospital (hours)	2.9 (1.0–8.8)	3.0 (1.0–8.7)	3.0 (1.0–9.2)	2.8 (1.0–8.4)	0.03
**Patients who used EMS**					
Symptom onset to call (min)	60 (12–288)	52 (10–261)	60 (12–282)	67 (15–349)	0.04
Call to EMS arrival (min)	6 (4–10)	7 (5–10)	5 (4–8)	6 (4–10)	<0.001
Call to hospital arrival (min)	32 (24–48)	33 (25–46)	30 (23–40)	40 (26–66)	<0.001

Abbreviations: SES—socioeconomic status; EMS—emergency medical service. Variables were reported as median and interquartile range. *p*-values were calculated using the =Kruskal-Wallis test.

**Table 3:** Multilevel multivariable logistic regression analysis for prehospital delay.

Variable	Unadjusted			Basic		Model 1			Model 2			Model 3		
	OR	95% CI	*p*	Model	*p*	aOR	95% CI	*p*	aOR	95% CI	*p*	aOR	95% CI	*p*
*Fixed effects*														
**Area-level SES** (Affluent)	1								1			1		
Middle	1.06	(0.98–1.16)	0.14						1.01	(0.85–1.18)	0.95	1.04	(0.90–1.20)	0.62
Deprived	1.27	(1.17–1.38)	<0.01						1.25	(1.08–1.45)	0.003	1.03	(0.89–1.20)	0.7
**Individual-level SES**														
**High school graduate** (below high school)	0.88	(0.82–0.95)	<0.01			0.91	(0.84–0.98)	0.02	0.92	(0.86–1.00)	0.04	0.93	(0.85–1.32)	0.07
**Occupation** (non-manual)	1					1			1			1		
Manual	1.14	(0.95–1.36)	0.15			1.09	(0.90–1.30)	0.39	1.08	(0.90–1.29)	0.4299	1.09	(0.90–1.32)	0.39
Other	1.00	(0.83–1.21)	1.00			0.95	(0.78–1.16)	0.61	0.96	(0.78–1.16)	0.64	1.04	(0.84–1.27)	0.73
Inactive	1.12	(0.94–1.33)	0.20			1.07	(0.90–1.28)	0.44	1.07	(0.90–1.29)	0.44	1.17	(0.97–1.42)	0.11
**National Health Insurance** (Medicaid)	0.99	(0.84–1.16)	0.88			1.00	(0.85–1.18)	1.00	1.01	(0.86–1.18)	0.93	0.94	(0.79–1.11)	0.45
*Covariates*														
**EMS use** (no)	0.32	(0.30–0.35)	<0.001									0.39	(0.36–0.42)	<0.001
**Interhospital transfer** (no)	2.29	(2.11–2.48)	<0.001									1.85	(1.69–2.03)	<0.001
**Age group (years)** (19–39)	1											1		
40–65	1.12	(0.90–1.40)	0.30									1.13	(0.90–1.43)	0.31
≥65	1.17	(0.94–1.45)	0.15									1.29	(1.01–1.63)	0.04
**Male** (female)	0.99	(0.92–1.06)	0.70											
**Previous diabetes** (no)	1.21	(1.12–1.31)	<0.001									1.25	(1.14–1.35)	<0.001
**Previous hypertension** (no)	1.01	(0.94–1.08)	0.76											
**Previous cardiovascular disease** (no)	0.79	(0.74–0.85)	<0.001									0.83	(0.77–0.90)	<0.001
**Exercise** (no)	0.88	(0.81–0.96)	<0.001									0.92	(0.83–1.01)	0.08
**Smoker** (never)	1													
Former	1.08	(1.00–1.18)	0.05											
Current	0.99	(0.90–1.09)	0.79											
**Alcohol consumption** (never)	1.05	(0.80–0.92)	0.21											
**Onset** (day)	0.86	(0.80–0.92)	<0.001									0.85	(0.79–0.92)	<0.001
**Loss of consciousness** (no)	0.55	(0.50–0.60)	<0.001									0.58	(0.52–0.64)	<0.001
**Recognition of symptoms** (no)	1.28	(1.17–1.40)	<0.001									1.04	(0.94–1.15)	0.48
**Urbanization**	0.74	(0.69–0.80)	<0.001									0.90	(0.78–1.04)	0.17
*Random effects*														
**Area-level variance**				0.147	<0.001	0.143		<0.001	0.133		<0.001	0.08		<0.001
**Intraclass correlation**				4.27		4.15			3.88			2.37		
														
**AUC**						0.599	(0.599–0.609)		0.599	(0.589–0.608)		0.693	(0.684–0.702)	
**AUC change**									<0.001			0.095		
														
*Model fitness*														
**AIC**				18,310.3		18,308.2			18,300.6			17,104.3		
**BIC**				18,317.1		18,332.2			18,331.4			17,172.7		

Abbreviations: SES—socioeconomic status; OR—odds ratio; CI—confidence interval; aOR—adjusted odds ratio; EMS—emergency medical service; AUC—area under curve; AIC—Akaike Information Criterion; BIC—Bayesian Information Criterion. All references are shown in parentheses. Basic—no predictor variables, random effect model; Model 1—basic model with individual-level SES included; Model 2—as per Model 1 but with area-level SES included; Model 3 = included both area-level and individual-level SES adjusted for age, exercise, diabetes, history of cardiovascular disease, stroke onset time, EMS use, transfer, loss of consciousness, recognition of symptoms, and urbanization status.

**Table 4:** Multivariate logistic regression analysis of prehospital delay stratified by urbanization status.

Variable	Urban			Rural		
	aOR	95% CI	*p*	aOR	95% CI	*p*
*Fixed effects*						
**Area-level SES** (Affluent)	1			1		
Middle	1.06	(0.92–1.22)	0.41	1.26	(0.88–1.79)	0.21
Deprived	1.24	(1.04–1.47)	0.02	1.04	(0.78–1.38)	0.81
**Individual-level SES**						
**High school graduate** (below high school)	0.92	(0.83–1.01)	0.09	0.95	(0.81–1.11)	0.49
**Occupation** (non-manual)	1			1		
Manual	1.25	(1.00–1.56)	0.048	0.68	(0.45–1.02)	0.06
Other	1.21	(0.95–1.53)	0.12	0.64	(0.41–0.99)	0.046
Inactive	1.35	(1.08–1.69)	0.01	0.72	(0.47–1.08)	0.11
**National Health Insurance** (Medicaid)	1.03	(0.82–1.28)	0.83	0.83	(0.64–1.07)	0.15
*Covariates*						
**EMS use** (no)	0.38	(0.35–0.42)	<0.001	0.42	(0.37–0.49)	<0.001
**Interhospital transfer** (no)	2.12	(1.86–2.42)	<0.001	1.56	(1.36–1.78)	<0.001
**Age group (years)** (19–40)	1			1		
40–65	1.12	(0.85–1.48)	0.44	1.17	(0.76–1.81)	0.48
≥65	1.22	(0.92–1.62)	0.18	1.46	(0.94–2.28)	0.09
**Previous diabetes** (no)	1.28	(1.15–1.42)	<0.001	1.2	(1.04–1.39)	0.01
**Previous cardiovascular disease** (no)	0.84	(0.76–0.92)	<0.001	0.84	(0.73–0.95)	0.01
**Exercise** (no)	0.94	(0.84–1.06)	0.31	0.88	(0.73–1.05)	0.15
**Onset** (day)	0.76	(0.69–0.83)	<0.001	1.02	(0.90–1.16)	0.79
**Loss of consciousness** (no)	0.54	(0.47–0.62)	<0.001	0.63	(0.53–0.74)	<0.001
**Recognition of symptoms** (no)	1.12	(0.98–1.28)	0.1	0.89	(0.75–1.06)	0.19
*Random effects*						
**Area-level variance**		0.03	<0.05		0.16	<0.001
**Intraclass correlation**		0.76			4.57	

Abbreviations: SES—socioeconomic status; OR—odds ratio; CI—confidence interval; EMS—emergency medical service. All references are shown in parentheses. Area and individual-level SES data were adjusted for age group, exercise, diabetes, previous cardiovascular disease, stroke onset time, EMS use, transfer, loss of consciousness, and recognition of symptoms.

## Supplementary Materials

The following is available online at https://www.mdpi.com/1660-4601/17/20/7392/s1: Supplementary Table S1: The indicators and components of the deprivation index in Korea, 2010. Supplementary Table S2. Characteristics of patients by urbanization status. Supplementary Table S3. Prehospital time intervals by urbanization status.
