# Supplementary material for: Association between Area-Level Socioeconomic Deprivation and Prehospital Delay in Acute Ischemic Stroke Patients: An Ecological Study
Source: Int J Environ Res Public Health. 2020 Oct 11;17(20):7392. doi: 10.3390/ijerph17207392 (PMC7600419; doi:10.3390/ijerph17207392)
Supplement: Supplementary file 1 [file ijerph-17-07392-s001.pdf]

Supplementary Material

Supplementary Table S1. The indicators and components of the deprivation index in Korea, 2010.

Indicators	Definition	Component	
		**Material**	**Social**
**Single family**	Single-person households/total households	0.42	0.79
**Lack of car**	Heads of households without a car/total heads of households	0.13	0.94
**Poor residential environment**	Households with poor housing environment*/total households	0.86	0.37
**Non-apartment living household**	Households with residential type that is not apartment/total households	0.55	0.7
**Female household**	Female head of household/total heads of households	0.1	0.92
**Low level of education**	Less than high school graduation in population aged 30–64/total population aged 30–64	0.77	0.56
**Old people**	People over 65 year of age/total people	0.81	0.57
**Low social status**	Heads of households with low social status/total population aged 15–64 years old	0.85	0.49
**Marriage**	Divorced or bereaved people over the age of 15/total people over the age of 15 years old	0.65	0.71
**Male unemployment**	Unemployed among the economically active males aged 15–64/total economically active males aged 15–64 years old	−0.9	0.05
**House owner**	Self-housing owned household/total households	−0.91	−0.22
	Eigenvalue	8.04	1.64
	Explained variance, %	48	40
	Cumulative variance, %	48	88

Source: Korean census, 2010. Note: Poor housing environment if one of the following is satisfied: no Western style toilet and separate kitchen in the house; no separate water supply system; no hot water supply system or separate bathroom in the house; no Western style and separate toilet in the house. Using briquette boiler or inner hearth or conventional type heating facility.

Supplementary Table S2. Characteristics of patients by urbanization status.

Variable	Total		Urban		Rural		*p*
	N	%	*N*	%	*N*	%	
**Total**	13,637		8463		5174		
**Sex (male)**	5815	42.6	3520	41.6	2295	44.4	0.002
**Age group (years)**							<0.001
19–40	352	2.58	248	2.93	104	2.01	
40–65	4290	31.5	2867	33.9	1423	27.5	<0.001
≥65	8995	66.0	5348	63.2	3647	70.5	
**Educational level**							<0.001
Below high school	8228	60.3	4441	52.5	3787	73.2	
≥High school graduate	5409	39.7	4022	47.5	1387	26.8	
**Type of occupation**							<0.001
Non-manual	565	4.14	428	5.06	137	2.65	
Manual	3656	26.8	1990	23.5	1666	32.2	
Other	1834	13.4	1321	15.6	513	9.9	
Inactive	7582	55.6	4724	55.8	2858	55.2	
**Type of insurance**							<0.001

**National Health Insurance**	12,953	95.0	8090	95.6	4863	94.0	
**Medicaid**	684	5.02	373	4.41	311	6.01	
**EMS use**	9232	67.7	5327	62.9	3905	75.5	<0.001
**Interhospital transfer**	4052	29.7	1603	18.9	2449	47.3	<0.001
**Area-level SES**							<0.001
Affluent	4489	32.9	3637	43.0	852	16.5	
Middle	4615	33.8	3519	41.6	1096	21.2	
Deprived	4533	33.2	1307	15.4	3226	62.4	

Abbreviations: ED, emergency department; EMS, emergency medical service; SES, socioeconomic status. The *p*-values were calculated using the chi-squared test.

Supplementary Table S3. Prehospital time intervals by urbanization status.

	**Total**		**Urban**		**Rural**		*p*
	Median	***IQR***	***Median***	***IQR***	***Median***	***IQR***	
**Total subjects**							
Symptoms to final hospital (hours)	4.3	1.7–10.4	4.0	1.3–10.2	4.7	2.1–10.7	<.0001
**Patients who were transferred**							
Symptoms to first hospital (hours)	2.9	1.0–8.8	3.1	1.0–9.2	2.6	1.0–7.9	<.0001
**Patients who used EMS**							
Symptoms to call (min)	60	12–288	60	13–303	58	11–248	0.22
Call to EMS arrival (min)	6	4–10	6	4–8	7	5–12	<.0001
Call to hospital arrival (min)	32	24–48	30	24–39	51	33–75	<.0001

Abbreviations: IQR, interquartile range; EMS, emergency medical service; The *p*-values were calculated using the Wilcoxon rank-sum test.
